# Disrupting monotony during social isolation stress prevents early development of anxiety and depression like traits in male rats

**DOI:** 10.1186/s12868-015-0141-y

**Published:** 2015-02-14

**Authors:** Saroj Kumar Das, Kalpana Barhwal, Sunil Kumar Hota, Mahendra Kumar Thakur, Ravi Bihari Srivastava

**Affiliations:** Experimental Biology Division, Defence Institute of High Altitude Research, Defence Research Development Organisation, Leh-Ladakh, C/O- 56 APO, Jammu and Kashmir, 901205 India; Department of Zoology, Banaras Hindu University, Varanasi, Uttar Pradesh India

**Keywords:** Monotony, Social isolation stress, Anxiety, Depression, Serotonin, Novel objects

## Abstract

**Background:**

Although there have been several reports on social isolation induced mood alterations, the independent contribution of monotonous environment in mediating mood alterations has been less studied. In view of the above, the present study is aimed at investigating the relative contribution of monotony towards mood alterations during isolation stress. Monotony was induced in a specially designed isolation chamber in male Sprague-Dawley rats in the presence or absence of isolation by housing animals singly (SH) or in pairs (PH). Novel objects were introduced to disrupt monotony in singly housed animals (SHNO) or paired housed animals (PHNO). Behavioural alterations were assessed using Open field test (OFT), Elevated Plus Maze (EPM) and Forced Swim Test (FST). Neuro-morphological changes in the CA3 region of hippocampus were studied by cresyl violet and golgi-cox staining. Hippocampal serotonin and 5-hydroxy indole acetic acid (5-HIAA) levels were estimated along with the expression of phospho-insulin like growth factor-1 receptor (pIGF-1R) and phospho cyclic AMP response-element binding protein (pCREB). Serotonin was depleted by administering Para-chlorophenylalanine (PCPA) to a separate PH group (PHPCPA), PHNO group (PHNOPCPA) and SHNO group (SHNOPCPA) to determine the role of serotonin in mediating monotony induced emotional mal-adaptations.

**Results:**

The results showed anxiety and depression like traits in both PH and SH groups during behavioural test such as OFT, EPM and FST. Pyknosis along with decrease in apical dendritic arborization was observed in the CA3 region of SH group along with decrease in serotonin and reduced expression of pIGF-1R and pCREB. Disrupting monotony through intervention of novel objects in PHNO and SHNO groups ameliorated anxiety and depression like traits and augmented pIGF-1R along with increase in serotonin level. Depletion of hippocampal serotonin level by PCPA administration in PHNOPCPA and SHNOPCPA groups on the other hand resulted in altered mood state despite disruption of monotony by novel objects intervention.

**Conclusion:**

The findings of our study suggest that monotonous environment independently contributes to impairment in mood state and disrupting monotony by intervention of novel objects during social isolation prevents mood disorders and emotional maladaptation through up regulation of hippocampal pIGF-1R and increase in serotonin.

## Background

Chronic social stress has been associated with the pathophysiology of psychological disorders including depression [1]. Early life adversities such as maternal separation or social isolation from conspecifics, are reported to adversely affect brain development and adult behavior [2-4]. Isolation rearing of rats leads to long term effects on mood behaviour, neuromorphology and neurotransmitters functions [5-7]. However, most of these experiments on rats involved housing animals in individual cages from the first day of weaning for a minimum period of 21 days [8] and there are few reports focusing on adult social isolation after social rearing. The focus in previous studies has been primarily on the effect of lack of social interaction with littermates while the effect of monotony due to lack of novelty during social isolation on behavior remains less studied. Chronic absence of novelty, repetition of events for a considerable period of time and no change in inhabiting environment over time leads to “monotony”. Diversion in attention during social isolation on the other hand, has been reported to lower stress responsive hormones in singly housed male and female rats [9]. However, none of these studies addressed a basic question as to whether the mood alterations were due to monotony or due to lack of interaction with conspecifics through physical, visual, auditory or pheromonal stimuli.

The behavioral alterations associated with monotony during chronic isolation stress could be an outcome of morphological, biochemical and molecular changes in the brain. The hippocampus, in particular, has been shown to be highly sensitive to stress [10] and has been implicated in the pathogenesis of depression [11]. Previous studies have reported structural changes and dendritic remodeling in the CA3 region of hippocampus in rats subjected to chronic social stress [12]. In addition to synaptic changes, alterations in neurotransmitters in the hippocampus has also been implicated for depression like behaviour [13]. Behavioral studies were performed to assess anxiety and depression like traits in the animals.

In the present study, we aimed at investigating the effect of monotony stress on the mood state of adult male Sprague-Dawley rats. The animals were exposed to monotony along with social isolation stress in a specially designed isolation chamber that could even prevent pheromonal contact during the period of exposure. Monotony was interrupted during isolation through introduction of novel objects for 4 hours on a daily basis continued for 14 days of exposure. Our study is an attempt to determine whether it was the prolonged monotony during social isolation or the lack of social interaction with conspecifics during isolation that causes behavioral changes in rats. In addition to behavioural changes, the present study also aimed at investigating the effect of monotony on serotonin mediated signaling mechanisms that could contribute to the mood state of the animals.

## Methods

### Chemicals

All the analytical chemicals used in these experiments were procured from Sigma-Aldrich Chemicals (St. Louis, MO, USA), unless mentioned otherwise. HPLC grade serotonin and 5HIAA standard and para-chlorophenylalanine (PCPA) were purchased from Sigma-Aldrich, USA. The chemicals used in western blot were obtained from Bio-Rad Laboratories (Bio-Rad, Hercules, CA, USA). Polyclonal primary antibodies for pIGF-1R and pCREB along with secondary antibodies were procured from AbCam (Cambridge Plc., USA).

### Experimental design

All protocols followed in the present study were approved by the ethics committee of the institute according to the guidelines of “Committee for the Purpose of Control and Supervision of Experiments on Animals” of Govt. of India (Registration No.- 1715/GO/c/13CPCSEA vide file No.- DIHAR/IAEC/13/2014). Adult male Sprague-Dawley rats weighing approximately 200 ± 10 g were used for the experiments. Food pellets (Lipton pvt Ltd, India) and water was given *ad libitum*. The animals were housed in clean hygienic conditions in the animal house maintained at 12 h light:dark cycle. Temperature and humidity were maintained at 25-28°C and 60-65%, respectively. Animals (n = 72) were randomly divided into seven experimental groups as depicted in Table 1.

**Table 1:** **Experimental groups**

**Groups**	**Description**	**Novel object intervention**	**Drug administration**	**Purpose**
1 (n = 12)	Paired housed (Two rats/cage)	YES	None	Control group without isolation and monotony **(PHNO)**
2 (n = 12)	Paired housed (Two rats/cage)	NO	None	Without isolation and with Monotony **(PH)**
3 (n = 12)	Paired housed (Two rats/cage)	YES	PCPA: 300 mg/kg (i.p.) (Koe and Weissman, [14])	Without isolation and monotony + PCPA **(PHNOPCPA)**
4 (n = 12)	Paired housed (Two rats/cage)	NO	PCPA: 300 mg/kg (i.p.)	Without isolation and with Monotony + PCPA **(PHPCPA)**
5 (n = 08)	Singly housed isolated rat (one rat/cage)	NO	None	With Isolation and monotony **(SH)**
6 (n = 08)	Singly housed isolated rat (one rat/cage)	YES	None	With Isolation and without monotony **(SHNO)**
7 (n = 08)	Singly housed isolated rat (one rat/cage)	YES	PCPA: 300 mg/kg (i.p.)	With Isolation and without monotony + PCPA **(SHNOPCPA)**

PCPA: Para chlorophenylalanine (Tryptophan hydroxylase inhibitor).

The rats were subjected to isolation along with monotony stress (SH) by housing them singly without novel object intervention in a specially designed isolation chamber (Seven stars, New Delhi, India). Temperature and humidity in each compartment of the isolation chamber was maintained precisely at 28 ± 2°C and 55 ± 5%, respectively with 12 hours day-night cycle. High Efficiency Particulate Air (HEPA) filter capable of trapping pheromonal particles were incorporated at the inlet point of fresh air into the social isolation chamber. Exposure was continuous for 14 days except for a 10–15 min interval each day for replenishment of food and water, novel object intervention, drug administration and changing of the cage housing. To induce monotony stress without isolation, the rats were housed in pairs (2 rats/cage) without novel objects intervention (PH). Novel objects were introduced into the housing environment of cage in singly housed animals (SHNO) and paired housed animals (PHNO) as intervention to disrupt monotony stress. Animals of PHNO group were considered as control group as they were not subjected to isolation and monotony.

The novel objects comprised of 14 distinct objects that had no resemblance in shape and appearance with each other and to which the animals were never exposed. Novel object was introduced, one at a time, to the cages housing the animals for a duration of 4 h each day during day time. A separate novel object was used each day and none of the novel object was repeated during the 14 days duration of stress exposure. The novel objects were introduced in a fixed area inside the cage and placed in such a way that they did not affect the space for the movement of the rats. The behavioural activities like licking, self-grooming, jumping, etc of the animals on introduction of the novel object were video recorded during the experiment.

### Drug preparation and pharmacological administration

Para-chlorophenylalanine (PCPA), a tryptophan hydroxylase inhibitor to deplete serotonin was dissolved in physiological saline (PS) and administered at a dose of 300 mg/kg BW on 0 day, 6^th^ day and 12^th^ day of exposure [14]. PCPA was administered intraperitoneally to the three experimental groups viz., PHNOPCPA, PHPCPA and SHNOPCPA. Equal volume of PS was administered intraperitoneally to rats of vehicle treated groups viz., PHNO, PH, SH and SHNO.

### Behavioural analysis

Rats (N = 79) were subjected to behavioural tests for exploratory, anxiety and depression like behaviour prior to and immediately after exposure to monotony stress. Baseline behavioural tests were conducted prior to stress exposure and 07 rats showing abnormal behaviour such as anxiety, depression and hyperactivity were excluded. A total of 72 male rats were then randomly divided into 8 experimental groups viz. PHNO, PH, PHNOPCPA, PHPCPA, SH, SHNO, SHNOPCPA. All behavioural experiments were carried out between 10.00 AM and 1.00 PM in standard conditions. Since Forced Swim Test (FST) is reported to induce depression like behavioural changes [15], the rats were first subjected to Open Field Test (OFT) followed by Elevated Plus Maze (EPM) and Forced Swim Test. Behavioural tests were then repeated after the period of exposure of 14 days for animals of all the experimental groups.

#### Open field test

Open field test was performed to assess the locomotory and exploratory behaviour of rats [16]. The open field maze comprised of two zones, central and peripheral zone which were delineated by a white line on the maze. The apparatus consisted of a rectangular area of 81 × 81 cm surrounded by a 28 cm high wall and lux intensity was maintained at 130-325 lux [17]. The rat was placed in one designated corner of the open field and its activity during the subsequent 5 min was assessed using ANY-maze software (Stoelting Co, USA). Horizontal locomotion, number of crossing over to the central zone and time spent in the peripheral and central zones were calculated and expressed as mean ± SEM.

#### Elevated plus maze test

Anxiety like behavior of rats was assessed by elevated plus maze test. It consisted of a plus-shaped apparatus with two opposing open arms (45 × 10 cm) and two enclosed arms (45 × 10 × 50 cm) elevated 65 cm from the floor and placed in a room with 500 lux intensity of light [18]. Rats were placed at the junction of the open and closed arms, facing the open arm opposite to the experimenter. The rats were allowed to move freely for a period of 5 min during which the number of entries and time spent by the rat in the open and closed arms was recorded by a video-tracking system and analyzed using the ANY-maze software (Stoelting Co, USA). Elevated plus maze was cleaned properly and dried with paper towel prior to each test to remove pheromonal traces from the excreta of the animals previously used in the same apparatus. The time spent (seconds) in the open and close arms and number of entries into the open and close arms were calculated to determine anxiety like behavior in the test animals.

#### Forced swim test

Depression like behavior in the animals was assessed by forced swim test (FST) [19]. In brief, the rats were released individually into a vertical transparent plexiglas cylinder (40 cm high; 20 cm in diameter) filled with 30 cm-deep water (25–30°C). Water was decanted and the glass cylinder was cleaned with 70% ethanol, dried and refilled with fresh water prior to each trial of animal during FST. The behavioral data was acquired by video-tracking system and analyzed using ANY maze software (Stoelting Co, USA). Behavioural scoring was performed by recording the struggling, swimming and floating time using the software. The struggling time provided an index of normal behavior as the rat tries to escape from the depressive situation.

### Sample preparation and analysis

For histological studies, rats were anesthetized with sodium pentobarbital and perfused intracardially with ice-cold 0.1 M phosphate buffer saline (PBS) followed by 4% paraformaldehyde, as suggested by Hota et al., [20]. Brain samples were cryoprotected in 30% sucrose in PBS for 48 h before serial cryosectioning. For biochemical and molecular estimations, rats were sacrificed by cervical dislocation and the brain was dissected at 4°C. The hippocampi were isolated and snap frozen in liquid nitrogen. The sample was then stored at -80°C till further analysis.

### Estimation of hippocampal serotonin and 5-HIAA level by HPLC

Quantification of serotonin (5-HT) and 5-hydroxy indole acetic acid (5-HIAA) in the hippocampal tissue were performed using high performance liquid chromatography (HPLC) with electrochemical detector as suggested by Mohanakumar et al., [21] using N-methylserotonin as internal standard. Briefly, 100 mg of hippocampal tissue was deproteinated at 4°C with 0.1 M perchloric acid containing 0.05% EDTA (1:10 dilution) followed by sonication and centrifugation at 10, 000 × g for 10 min at 4°C. The supernatant was filtered through syringe filters (0.22 μm, Millipore) and filtrate (10 μl) was injected directly into an HPLC system (Waters Corporation, USA) with C_18_ analytical column (4.6 cm × 25 cm) using auto-sampler. The mobile phase contained 8.65 mMol heptane sulphonic acid/lit, 0.27 mMol EDTA/lit, 13% (v/v) acetonitrile, 0.4–0.45% (v/v) triethylamine and 0.20–0.25% (v/v) phosphoric acid. The flow rate was maintained at 0.7 ml/min and the electro- detection was performed at 0.74 V. The data were collected and integrated in a Waters 745B data module. Quantification of serotonin and 5-HIAA in samples was carried out using standard plot of serotonin and 5-HIAA and the results were expressed in ng/mg of wet tissue samples.

### Histological studies

#### Assessment of morphological alterations in the CA3 region by cresyl violet staining

Cresyl violet staining was performed according to the method suggested by Hota et al., [22]. Serial frozen sections of 15 μm thickness were obtained from cryoprotected brain (n = 6 per group) and sections corresponding to bregma −4.16 mm were used for cresyl violet staining. Sections were stained with 0.1% cresyl violet (in 3% acetic acid) for 15 min followed by washing with distilled water. Sections were then dehydrated in serial dilutions of ethanol ranging from 50 to 100% and cleared with xylene. The sections were mounted with DPX and the CA3 region was visualized under a bright field microscope (Olympus, CX31, Japan). Six serial sections were taken from each brain sample and neurons showing staining pattern characteristic of pyknosis were counted in an area of 0.1 mm^2^ in six different views of hippocampal CA3 region in each section using Stereo Investigator software (MBF Bioscience, USA). The results were expressed as mean values.

#### Quantification of apical dendritic arborization by golgi-cox staining

Golgi-Cox staining was performed according to the protocol suggested by Hota et al., [23]. In brief, animals were perfused with normal saline and the brain was removed and placed in Golgi-Cox stain (5% potassium dichromate solution, 5% mercuric chloride, 5% potassium chromate and distilled water in the ratio 5:5:4:10) and stored in dark for 15 days. The brain was then cryoprotected in ice-cold 30% sucrose solution and sectioned using vibratome (Leica-VT1000S, Germany). Sections (200 μm thickness) were collected in 6% sucrose solution and transferred to gelatin coated slides. Slides were then washed in distilled water, immersed in ammonium hydroxide for 30 min, washed and then immersed in Kodak fix solution for 30 min in a dark room. Slides were washed and dehydrated with serial dilutions of alcohol and finally kept in a solution of chloroform, xylene and 100% alcohol (ratio 1:1:1) for 15 min. Slides were then mounted with DPX and visualized under a bright field microscope (Olympus, CX31, Japan). The apical dendritic arborization was quantified using the neurolucida software and were expressed in percentage by considering mean value of PHNO group to be 100%.

### Protein estimation

The protein content of all the samples were estimated by the method suggested by Bradford [24] using BSA as the standard.

### Expression of pIGF-1R and pCREB in hippocampus by western blotting

The hippocampi were dissected out at 4°C and homogenized in ice-cold lysis buffer (0.01 M Tris–HCl, 0.1% NaN3, 0.1 M NaCl, 0.1 M dithiothreitol, 1 mM EDTA, 100 μg/mL PMSF, protease inhibitor cocktail, (pH 7.6)). Separation of cytosolic and nuclear fraction was carried out as per the protocol suggested by Barhwal et al., [25]. Sample protein (50 μg) was resolved by 10% SDS-PAGE and transferred to nitrocellulose membranes pre-soaked in transfer buffer (20% methanol, 0.3% Tris–HCl, 1.44% glycine) using a semidry transblot module (Bio-Rad, Hercules, CA, USA). The transfer of the protein bands to the membrane was verified by Ponceau staining. The membrane was then blocked with 5% Blotto (Sigma Aldrich, USA) for 1 h and washed with 0.01 M phosphate buffer saline, pH 7.4, 0.1% Tween 20 (PBST). The membranes were incubated overnight with polyclonal pIGF-1R and pCREB antibodies (AbCam, Cambridge Plc., USA). Subsequently, the membranes were washed with PBST thrice for 10 min each and incubated with secondary anti-IgG HRP conjugated antibody for 2–3 h. The membranes were then developed using chemiluminiscent peroxidase substrate kit (Sigma Aldrich, USA), stripped using stripping buffer (Bio-Rad, Hercules, CA, USA) and probed for β-actin expression. The protein expression was quantified by densitometry and were expressed in percentage by considering mean value of PHNO group to be 100%.

### Statistical analysis

The mean and standard error of mean were calculated for each group. One way ANOVA was performed and post hoc analysis was done by Tukey’s test using SPSS software. Difference below or equal to the probability level 0.01 was considered statistically significant.

## Results

### Behavioural assessment

#### Open field test

**Figure 1:**
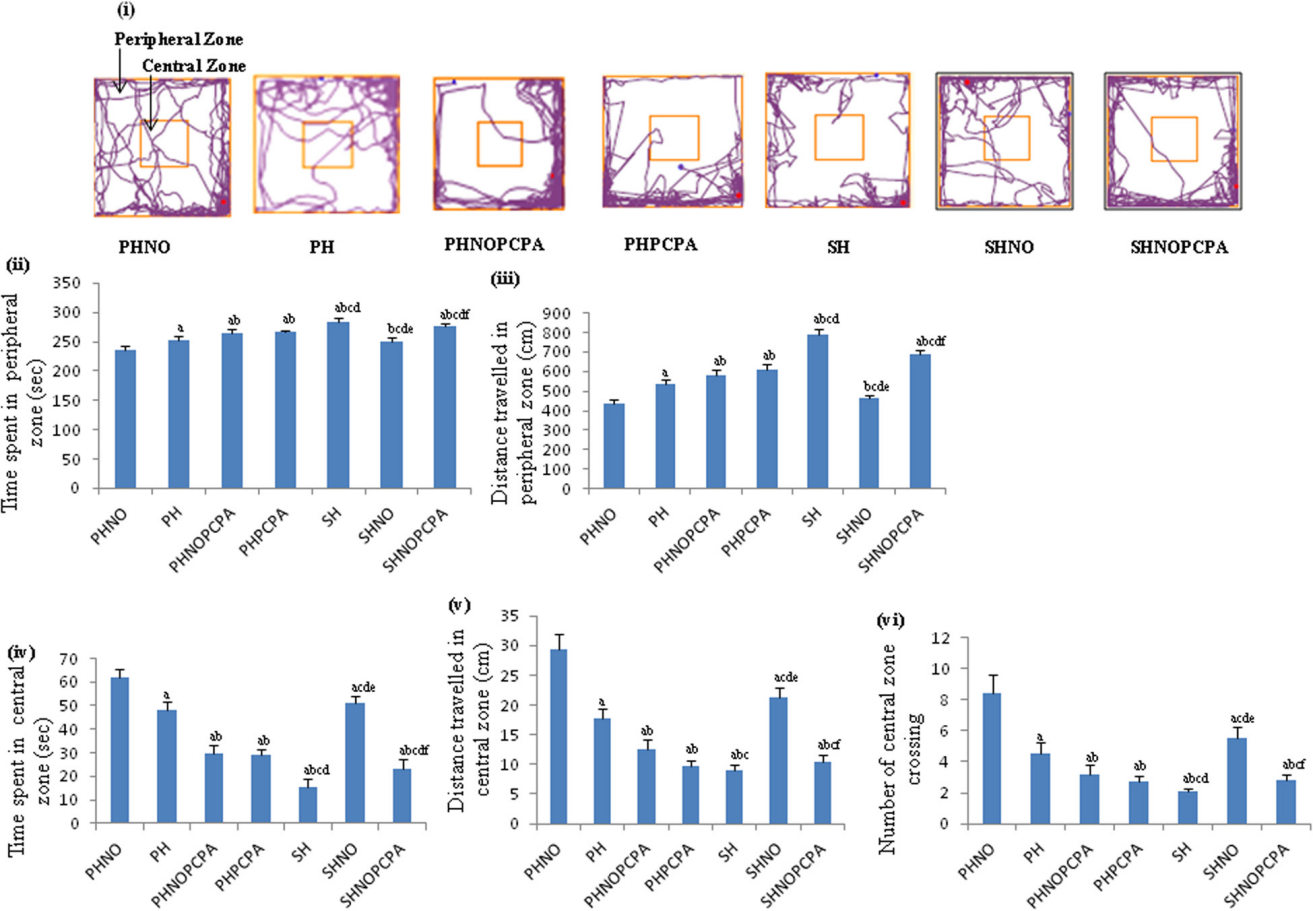
**Open field test. i)** Representative tracksheets of open field test and graphs showing alterations in **(ii)** time spent in peripheral zone, **(iii)** distance travelled in peripheral zone, **(iv)** time spent in central zone, **(v)** distance travelled in central zone and **(vi)** number of central zone crossing following exposure to monotony stress, PCPA administration and novel object intervention during isolation. Values are expressed as mean ± SEM. ‘a’ denotes p ≤ 0.01 when compared to PHNO group, ‘b’ denotes p ≤ 0.01 when compared to PH, ‘c’ denotes p ≤ 0.01 when compared to PHNOPCPA group, ‘d’ denotes p ≤ 0.01 when compared to PHPCPA group and ‘e’ denotes p ≤ 0.01 when compared to SH group and ‘f’ denotes p ≤ 0.01 when compared with SHNO group.

Open field test showed that rats in the SH group, subjected to both isolation and monotony stress spent more time in the peripheral zone when compared with SHNO and PH groups (*F*_6,65_ = 63.51, *P* < 0.01) as depicted in the representative track sheet (Figure 1i). The SHNOPCPA group on the other hand, registered significant decline in distance travelled in central zone (*F*_6,65_ = 228.2, *P* < 0.01) (Figure 1v), time spent in central zone (*F*_6,65_ = 288.4, *P* < 0.01) (Figure 1iv) and central zone crossing (*F*_6,65_ = 104.6, *P* < 0.01) (Figure 1vi) along with concomitant increase in distance travelled in peripheral zone (*F*_6,65_ = 236.8, *P* < 0.01) (Figure 1iii) and time spent in peripheral zone (*F*_6,65_ = 63.51, *P* < 0.01) (Figure 1ii) as compared to PHNO group. The SH group also spent more time in peripheral zone (Figure 1ii) along with decrease in time spent in central zone (Figure 1iv) when compared to PHNO group. SHNO group, on the other hand, registered significant enhancement in the time spent in central zone (Figure 1iv) and frequency of central zone crossing (Figure 1vi) along with decrease in the time spent in peripheral zone when compared to SH group (Figure 1vi). PCPA administration in PHNOPCPA and PHPCPA groups also significantly reduced the time spent in central zone (*F*_6,65_ = 288.4, *P* < 0.01) (Figure 1iv) and central zone crossing (*F*_6,65_ = 104.6, *P* < 0.01) (Figure 1vi) as compared to PHNO and PH groups respectively.

#### Elevated plus maze

**Figure 2:**
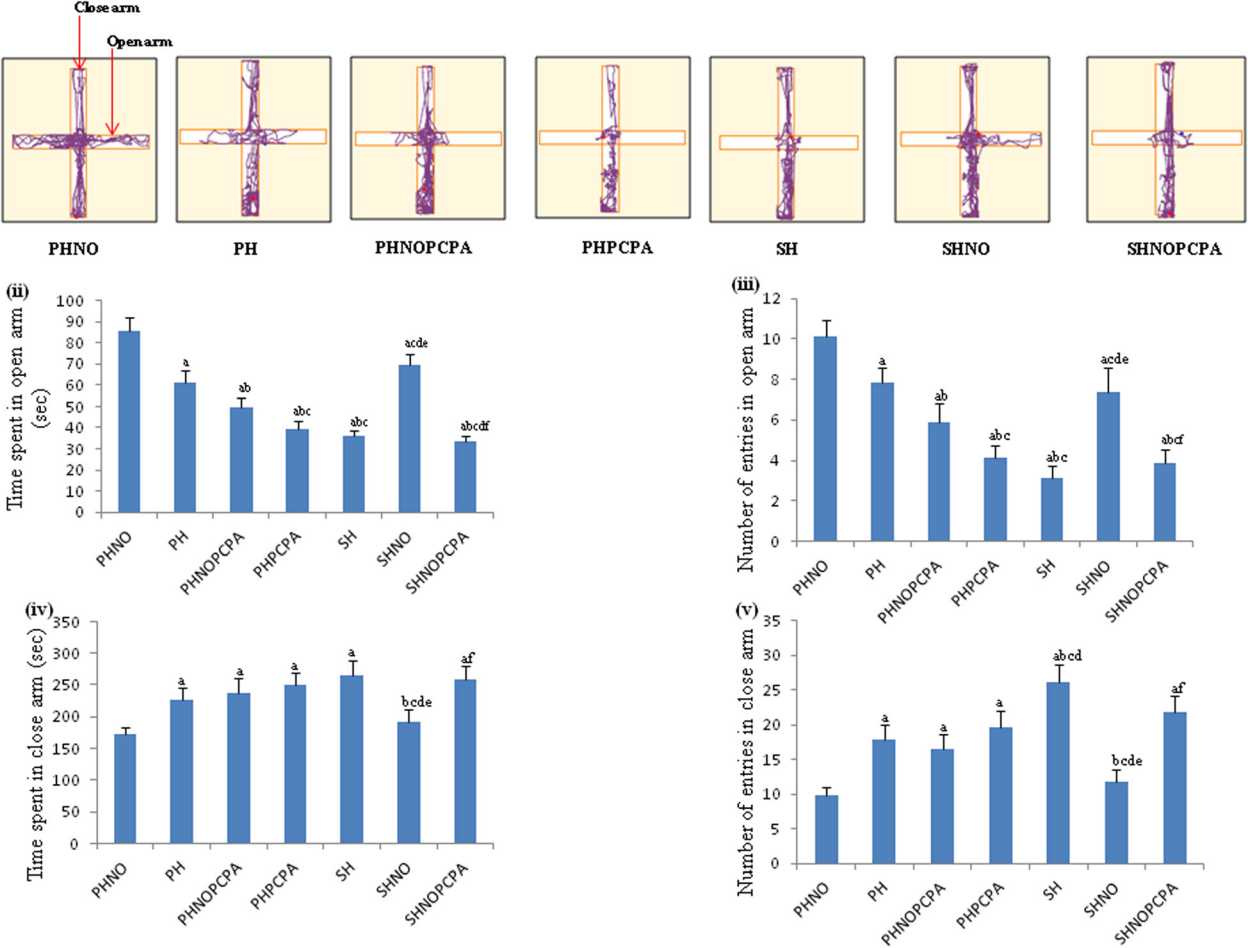
**Elevated plus maze test. i)** Representative tracksheets of elevated plus maze test. Graphs showing alterations in **(ii)** time spent in open arm, **(iii)** number of entries in open arm, **(iv)** time spent in close arm and **(v)** number of entries in close arm in elevated plus maze following exposure to monotony stress, PCPA administration and novel object intervention during isolation. Values are expressed as mean ± SEM. ‘a’ denotes p ≤ 0.01 when compared to PHNO group, ‘b’ denotes p ≤ 0.01 when compared to PH, ‘c’ denotes p ≤ 0.01 when compared to PHNOPCPA group, ‘d’ denotes p ≤ 0.01 when compared to PHPCPA group and ‘e’ denotes p ≤ 0.01 when compared to SH group and ‘f’ denotes p ≤ 0.01 when compared with SHNO group.

There was a significant decrease in the time spent in the open arm (*F*_6,65_ = 174.9, *P* < 0.01) (Figure 2ii) and number of open arm entries (*F*_6,65_ = 105.3, *P* < 0.01) (Figure 2iii) with the concomitant increase in time spent in close arm (*F*_6,65_ = 31.64, *P* < 0.01) (Figure 2iv) and number of close arm entries (*F*_6,65_ = 64.55, *P* < 0.01) (Figure 2v) in the SH group when compared to PHNO. On the contrary, the number of entries to open arm (*F*_6,65_ = 105.3, *P* < 0.01)as well as time spent in open arm (*F*_6,65_ = 174.9, *P* < 0.01)was significantly higher in SHNO group when compared to SH group. In comparison to PHNO and SHNO groups, the PH group showed significant decrease in number of open arm entries and time spent in the open arm. PHNOPCPA and SHNOPCPA groups also showed significant decrease in open arm entries (Figure 2iii) and increase in close arm entries when compared to PHNO and SHNO groups (Figure 2v).

#### Forced swim test

**Figure 3:**
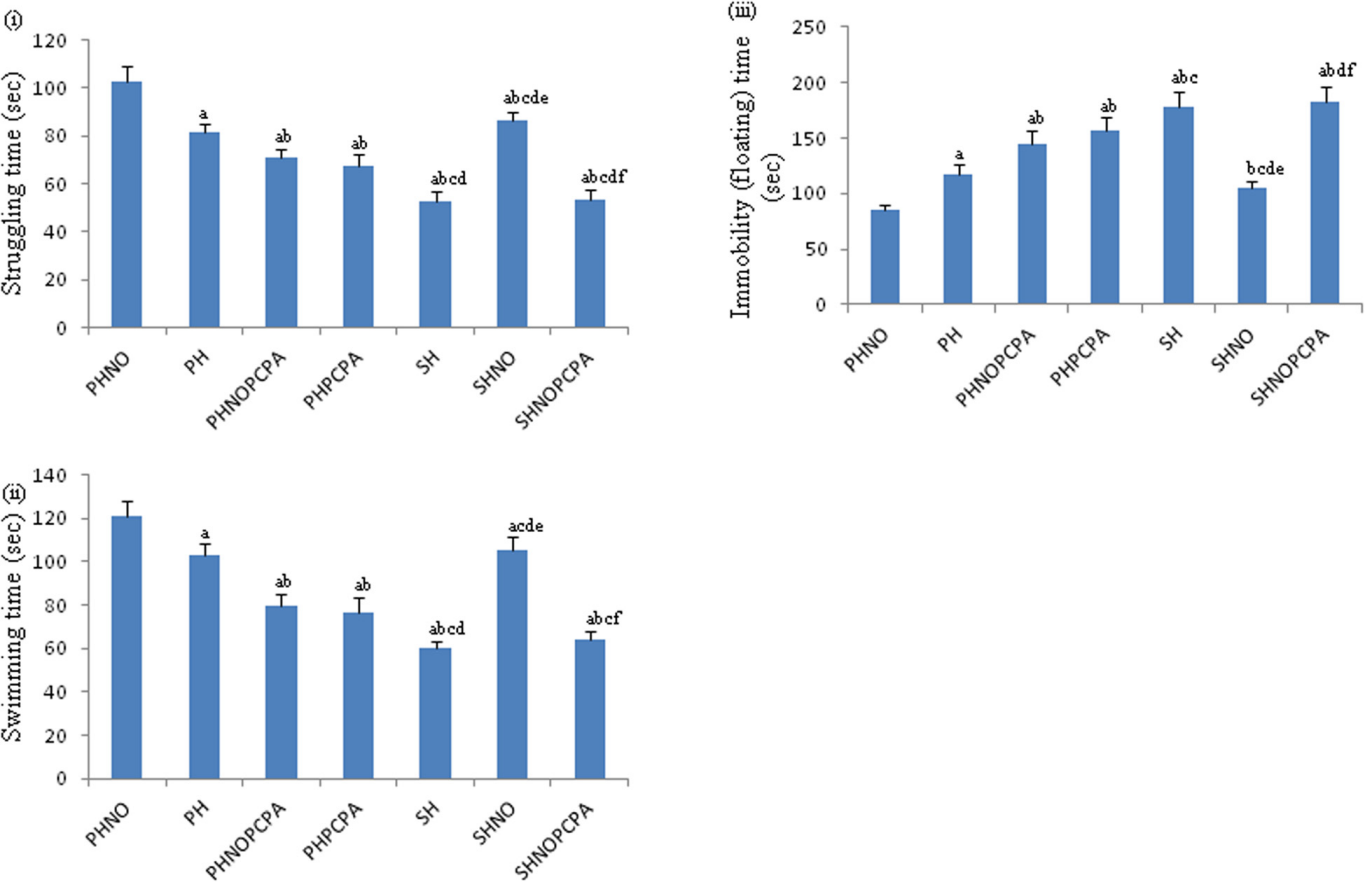
**Force swim test.** Graphs representing changes in **(i)** struggling time, **(ii)** swimming time and **(iii)** immobility (floating) time in Force swim test following exposure to monotony stress, PCPA administration and novel object intervention during isolation. Values are expressed as mean ± SEM. ‘a’ denotes p ≤ 0.01 when compared to PHNO group, ‘b’ denotes p ≤ 0.01 when compared to PH, ‘c’ denotes p ≤ 0.01 when compared to PHNOPCPA group, ‘d’ denotes p ≤ 0.01 when compared to PHPCPA group and ‘e’ denotes p ≤ 0.01 when compared to SH group and ‘f’ denotes p ≤ 0.01 when compared with SHNO group.

Increase in floating (immobility) time (*F*_6,65_ = 115.6, *P* < 0.01) (Figure 3iii) as well as decrease in the swimming time (*F*_6,65_ = 158.2, *P* < 0.01) (Figure 3ii) was observed in SH group during forced swim test (FST) when compared to PHNO group. There was a significant decrease in immobility time (*F*_6,65_ = 115.6, *P* < 0.01) (Figure 3iii) with concomitant increase in struggling time (*F*_6,65_ = 179.1, *P* < 0.01) (Figure 3iii) in SHNO group when compared to SH and PH groups. PCPA administration in PHNOPCPA and PHPCPA groups also significantly reduced the immobility time (*F*_6,65_ = 115.6, *P* < 0.01) (Figure 3iii) with the corresponding decrease in the struggling time (*F*_6,65_ = 197.1, *P* < 0.01) (Figure 3i) when compared with PHNO and PH groups respectively.

### Changes in the serotonin (5-HT) and 5-HIAA level

**Figure 4:**
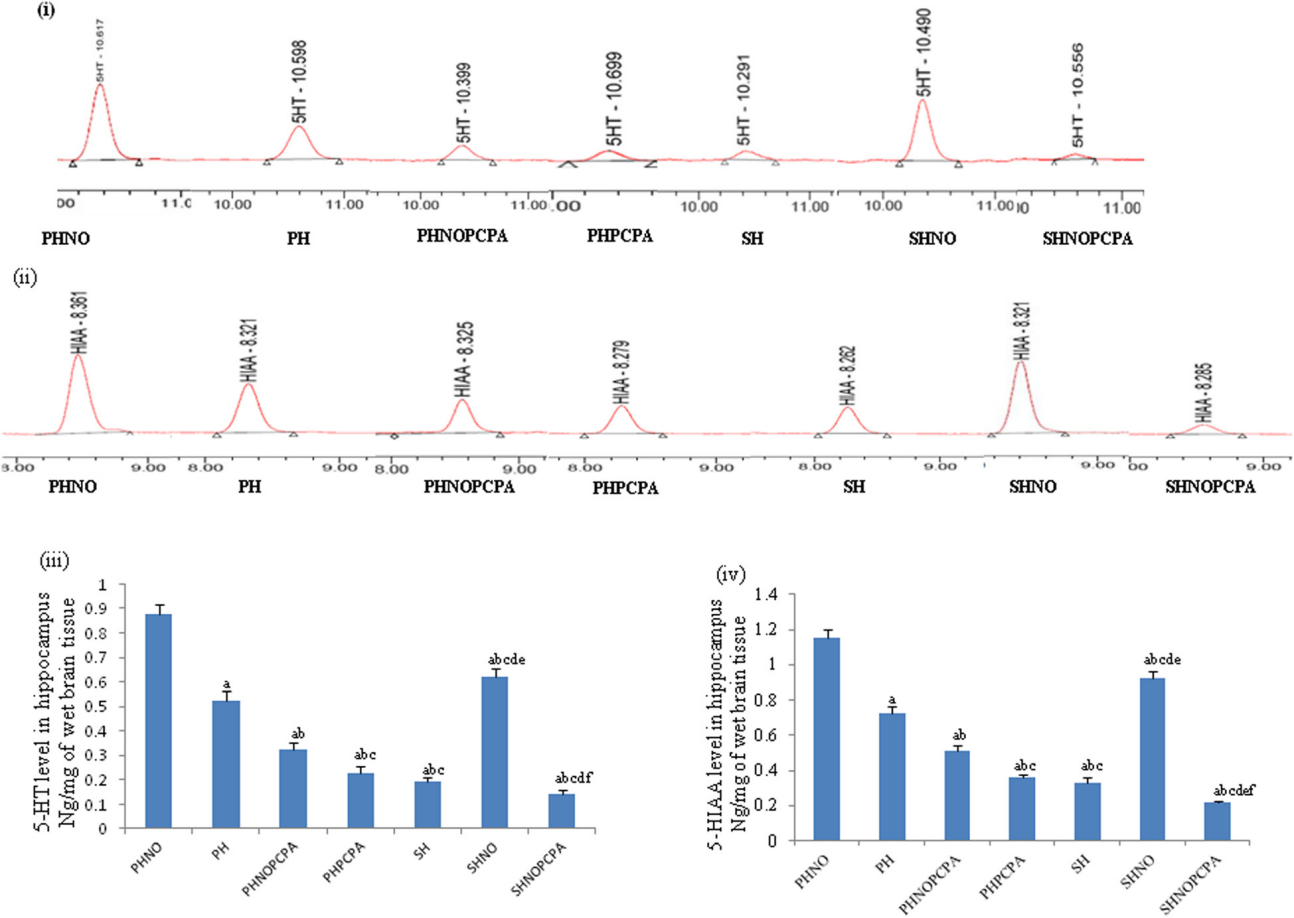
**Estimation of serotonin (5-HT) and 5-hydroxy indole acetic acid (5-HIAA) by HPLC. i)** Representative chromatograms showing the peaks for serotonin (5-HT), **(ii)** representative chromatograms showing the peaks for 5-HIAA, graph showing **(iii)** changes in the level of 5-HT and **(iv)** changes in the level of 5-HIAA in hippocampus following exposure to monotony stress, PCPA administration and novel object intervention during isolation. Values are expressed as mean ± SEM. ‘a’ denotes p ≤ 0.01 when compared to PHNO group, ‘b’ denotes p ≤ 0.01 when compared to PH, ‘c’ denotes p ≤ 0.01 when compared to PHNOPCPA group, ‘d’ denotes p ≤ 0.01 when compared to PHPCPA group and ‘e’ denotes p ≤ 0.01 when compared to SH group and ‘f’ denotes p ≤ 0.01 when compared with SHNO group.

Estimation of 5-HT (*F*_6,29_ = 325.4, *P* < 0.01) (Figure 4iii) and 5-HIAA (*F*_6,29_ = 506.9, *P* < 0.01) (Figure 4iv) level in hippocampal region showed a significant decrease in SH group as compared to SHNO group. SHNOPCPA group showed significant decrease in hippocampal serotonin level when compared with SHNO group (*F*_6,29_ = 325.4, *P* < 0.01) as shown in Figure 4iii. SHNOPCPA group showed increased anxiety and depression like traits due to depletion of central serotonin level with a concomitant decrease in 5-HIAA level as compared to PHNO and PH groups (Figure 4iii and iv). PH group showed significant decrease in serotonin levels in the hippocampus as compared to PHNO group (*F*_6,29_ = 325.4, *P* < 0.01). PCPA administration in PHNOPCPA and PHPCPA groups showed significant decrease in the 5-HT (*F*_6,29_ = 325.4, *P* < 0.01) and its metabolite 5-HIAA (*F*_6,29_ = 506.9, *P* < 0.01) as compared to SHNO group.

### Histological studies

#### Pyknotic cell counts by cresyl violet staining

**Figure 5:**
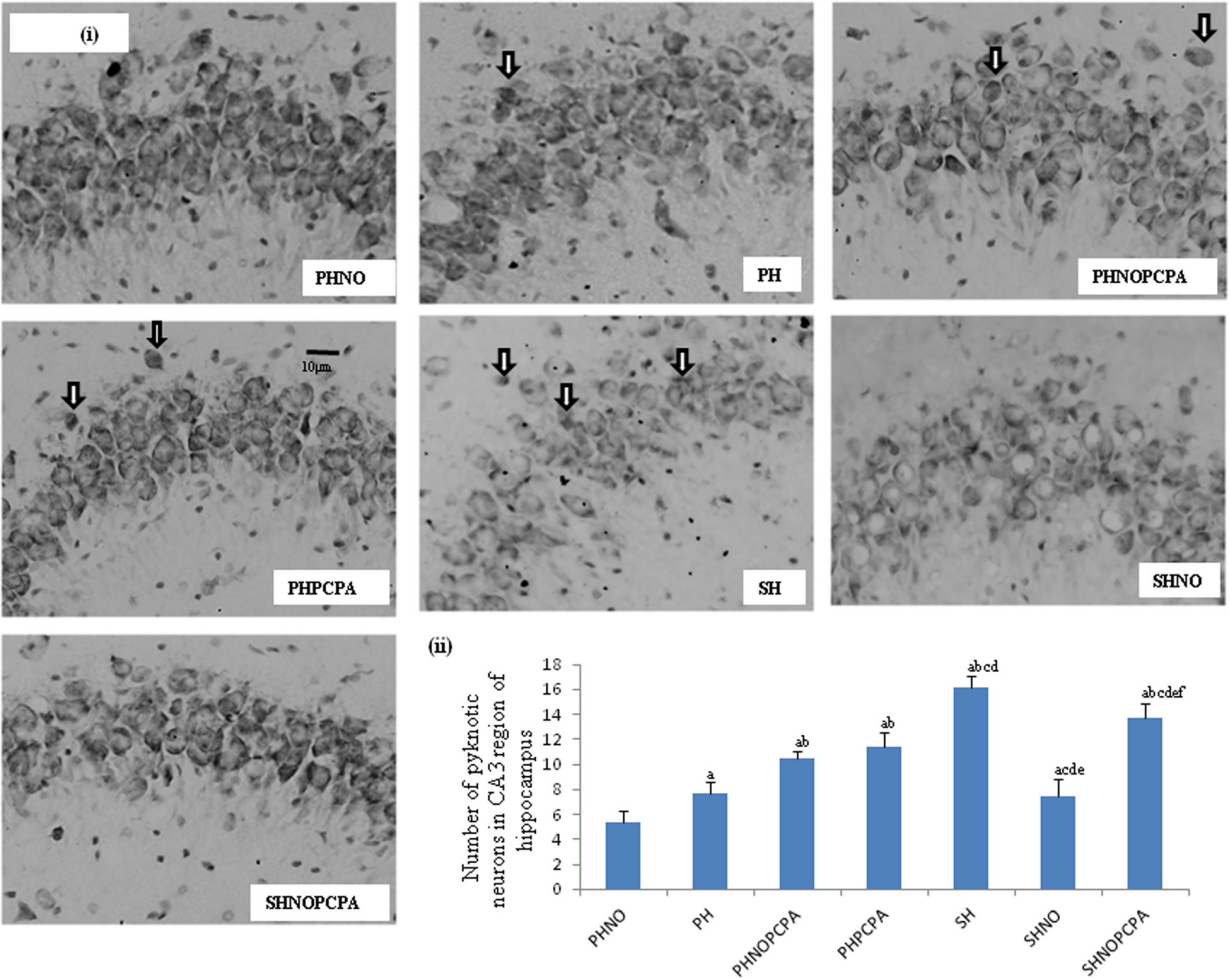
**Cresyl violet staining. i)** Representative images of cresyl violet staining depicting pyknotic cells in the CA3 region of the hippocampus. Arrow heads depict pyknotic cells. Scale bar-10 μm. **ii)** Graph showing changes in number of pyknotic neurons in the CA3 region of hippocampus following exposure to monotony stress, PCPA administration and novel object intervention during isolation. Values are expressed as mean ± SEM. ‘a’ denotes p ≤ 0.01 when compared to PHNO group, ‘b’ denotes p ≤ 0.01 when compared to PH, ‘c’ denotes p ≤ 0.01 when compared to PHNOPCPA group, ‘d’ denotes p ≤ 0.01 when compared to PHPCPA group and ‘e’ denotes p ≤ 0.01 when compared to SH group and ‘f’ denotes p ≤ 0.01 when compared with SHNO group.

The number of pyknotic neurons in the CA3 region of the hippocampus increased significantly in SH group as compared to PHNO group (*F*_6,29_ = 86.28, *P* < 0.01) (Figure 5ii). The neuronal pyknosis in SHNO group was significantly lower when compared to animals subjected to similar duration of exposure to isolation and monotony stress (SH) as shown in Figure 5ii (*F*_6,29_ = 86.28, *P* < 0.01). PCPA administration to experimental groups such as PHNOPCPA and PHPCPA showed significant increase in pyknotic neurons as compared to PHNO and PH group respectively (*F*_6,29_ = 86.28, *P* < 0.01).

#### Alterations in apical dendritic arborization by golgi-cox staining

**Figure 6:**
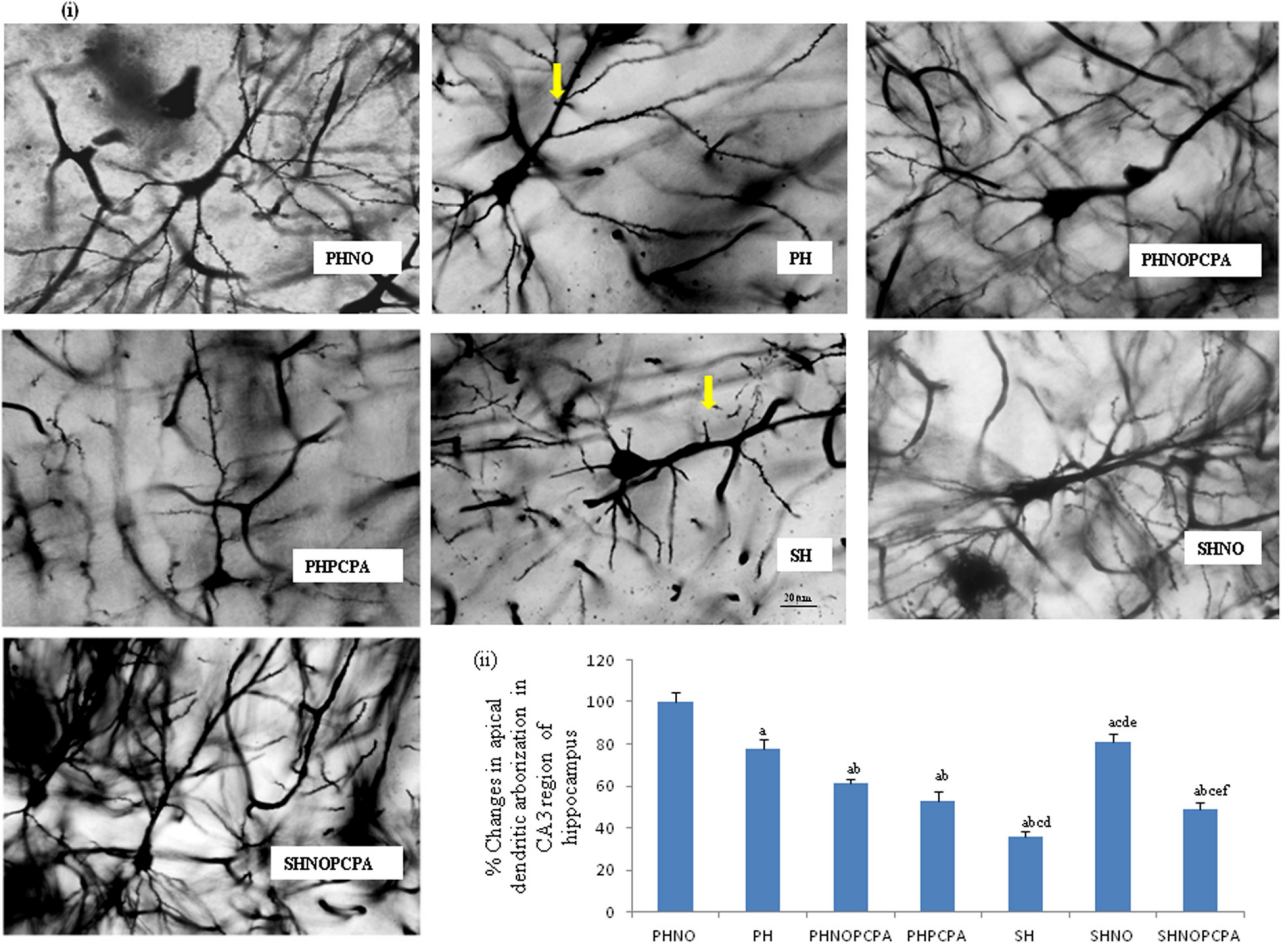
**Golgi-cox staining. i)** Representative micrographs of Golgi-cox staining showing dendritic arborization in the CA3 region of the hippocampus. Arrow heads depict loss of dendritic arborization. Scale bar-20 μm. Magnification-40X. **ii)** Graph showing percentage changes in apical dendritic arborization in CA3 region of the hippocampus following exposure to monotony stress, PCPA administration and novel object intervention during isolation considering mean value of PHNO group to be 100%. Values are expressed as mean ± SEM. ‘a’ denotes p ≤ 0.01 when compared to PHNO group, ‘b’ denotes p ≤ 0.01 when compared to PH, ‘c’ denotes p ≤ 0.01 when compared to PHNOPCPA group, ‘d’ denotes p ≤ 0.01 when compared to PHPCPA group and ‘e’ denotes p ≤ 0.01 when compared to SH group and ‘f’ denotes p ≤ 0.01 when compared with SHNO group.

Golgi-Cox staining showed significant decrease in apical dendritic arborization in the CA3 region of hippocampus of the SH group when compared to PHNO and PH groups considering mean value of PHNO group to be 100%. SHNO group showed significant increase in the apical dendritic arborization in the CA3 region of hippocampus compared to SH group as shown in Figure 6ii. Rats of experimental group who were housed in pair without novel objects intervention (PH) showed significant reduction in apical dendritic arborization in CA3 region of hippocampus as compared to PHNO group.

#### Changes in pIGF-1R and pCREB expression in hippocampus

**Figure 7:**
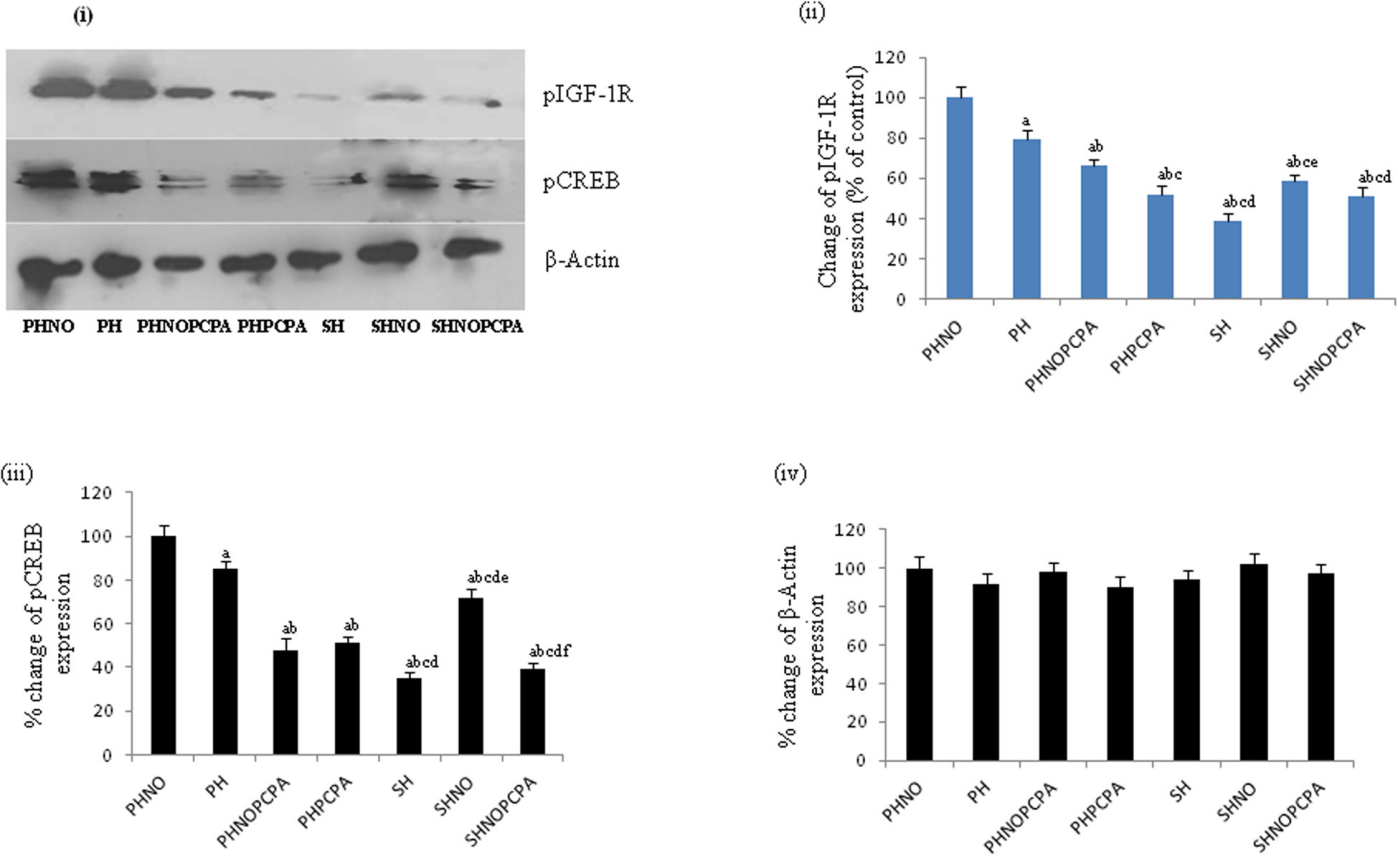
**Protein expression study by western blotting. i)** Representative western blots showing expression of pIGF-1R, pCREB and β-Actin. Densitometric results depict the changes in expression of **(ii)** pIGF-1R, **(iii)** pCREB and **(iv)** β-Actin following exposure to monotony stress, PCPA administration and novel object intervention during isolation. Values are expressed as percentage change in protein expression considering mean OD of PHNO group to be 100%. ‘a’ denotes p ≤ 0.01 when compared to PHNO group, ‘b’ denotes p ≤ 0.01 when compared to PH, ‘c’ denotes p ≤ 0.01 when compared to PHNOPCPA group, ‘d’ denotes p ≤ 0.01 when compared to PHPCPA group and ‘e’ denotes p ≤ 0.01 when compared to SH group and ‘f’ denotes p ≤ 0.01 when compared with SHNO group.

Rats in the SH group showed significant (p ≤ 0.01) decrease in the expression of pIGF-1R in the hippocampal region when compared with PHNO group (Figure 7ii). SH group showed a significant decrease in hippocampal pCREB expression as compared to PHNO group. Expression of pCREB in the hippocampus was significantly higher in the SHNO group as compared to SH group (Figure 7iii). However, in SHNOPCPA group, there was a marginal decrease in pIGF-1R expression as compared to SHNO group which was non significant (Figure 7ii).

## Discussion

Isolation causes altered mood state that often leads to manifestation of persistent anxiety, hyperactivity, aggressiveness and sometimes to depressive traits [26-30]. It has been previously reported that social isolation due to restriction of physical contact with conspecifics for 3 weeks or more induces depression like behaviour in rats [31]. While most of the research on isolation is focused on tactile and sensory isolation from conspecifics, the relative contribution of monotonous environment in inducing anxiety and depression like traits has been less studied. In the present study, we created an experimental model where the effect of monotony on mood state could be studied. Rats in the SH group were subjected to both isolation and monotony stress by housing them singly in the specially designed isolation chamber. In the SHNO group animals were subjected to social isolation stress in the isolation chamber but, monotony was disrupted through introduction of novel objects into the housing cages. The PH group on the other hand, comprised of two animals housed socially in the same cage without novel objects intervention thereby subjecting them to monotony in the absence of social isolation stress. The control group on the other hand comprised of two rats housed socially along with novel objects intervention (PHNO) and thus were not subjected to isolation and monotony. In contrast to previous studies that required the rats to be housed in isolation for 3 weeks, our study showed that the animals subjected to isolation and monotony stress (SH) in the specially designed isolation chamber that restricted tactile, pheromonal and visual stimuli showed anxiety and depression like traits at a shorter duration of 14 days in the isolation chamber [8].

The present study shows that monotony alone can mediate mood alterations independent of isolation. Rats in both SH and PH groups that were subjected to monotony stress showed anxiety and depression like traits as indicated by decrease in time spent in central zone in OFT, decrease in open arm entry in EPM and increase in floating time in FST. The predominant contribution of monotony during isolation towards mediating behavioural alterations is further supported by our results showing altered mood state in rats of PH group in which two rats were socially housed but without novel objects intervention. Disrupting monotony through introduction of novel objects on the other hand resulted in improvement in mood state in paired housed and singly housed rats resulting in amelioration of the anxiety and depressive traits as seen in PHNO and SHNO groups. Rats in the SHNO group showed decrease in anxiety and depression like traits despite being housed in isolation. This clearly indicates that monotony can independently induce early emotional mal-adaptation like anxiety and depressive traits irrespective of isolation.

Behavioural alterations due to isolation has been previously attributed to altered neurotransmitter metabolism in the hippocampus [32]. Decreased synaptic availability of serotonin, in particular, has been shown to mediate depressive traits in both experimental and clinical conditions [33]. The wide use of selective serotonin reuptake inhibitors (SSRIs) for treatment of depression in clinical psychiatry further establishes the role of serotonin in modulating the mood conditions [34]. We, therefore, estimated serotonin and its metabolite i.e., 5-hydroxy indole acetic acid (5-HIAA) concentration in the hippocampus and investigated the effect of para chloro phenyl alanine (PCPA) administration as serotonin depletor and to determine a correlation between monotony induced anxiety and depression like traits and serotonin concentration. Concomitant to earlier findings on depletion of serotonin in isolation induced depression, rats in the SH group as well as PH group showed decrease in serotonin in the hippocampus during the present study [35]. Conversely, disrupting monotony in the SHNO group resulted in elevation of serotonin concentration as compared to SH group. This increase in serotonin on introduction of novel objects during isolation could be due to activation of reward circuits and triggering the exploratory behavior in rats. Depletion of serotonin by administration of PCPA was able to induce anxiety as well as depression like behaviour in isolated rats despite introduction of novel objects as evident from the results of OFT, EPM and FST. This provides necessary evidence for a pivotal role of serotonin in novel object induced improvement of mood status and amelioration of depressive traits in rats. Our findings also find support from previous reports showing mood alterations due to decrease in availability of serotonin at the synaptic cleft [36,37].

In addition to behavioural changes and alteration in serotonin concentration in the hippocampus, we also observed increased neurodegeneration and neuronal pyknosis in the CA3 region of the hippocampus along with decrease in apical dendritic branching in rats subjected to monotony stress. The CA3 region has been previously reported to be innervated by serotonergic neurons. Studies by Kuramochi et al. [13] showed reduction in the number of 5-HT axons in the CA3 region of hippocampus during exposure to social isolation stress. The decrease in apical dendrites observed in the SH and PH groups during the present study may be attributed to the decreased serotonergic input to CA3 neurons. Similar observations on decreased hippocampal dendritic arborization in rats exposed to chronic social stress have been made by Mckittrick et al., [12]. Rats in SHNO group with novel object intervention on the other hand showed higher dendritic arborization with decreased pyknosis in hippocampal CA3 region when compared to rats reared in monotony stress (PH and SH).

Previous studies on enriched environment have shown improvement in exploratory behaviour and occurrence of neurogenesis in rat brain. Studies by Wadowska et al., [38] have shown improvement in IGF-1 signalling when the rats were housed in enriched environment following transient global cerebral ischemia. IGF-1 has also been suggested to be a as possible target for therapy in depression [39]. Studies by Hoshaw et al., [40] showed that administration of IGF-1 increased basal serotonin levels in the ventral hippocampus. We, therefore, investigated on a possible role of IGF-1R mediated mechanism for increase in serotonin on disrupting monotony through novel object intervention. Our findings show decrease in expression of pIGF-1R in the PH and SH groups when compared to control group (PHNO), while disrupting monotony through intervention of novel objects in the SHNO group resulted in increased phosphorylation of IGF-1R when compared to SH group. Our findings on pIGF-1R mediated increase in concentration of serotonin find support from studies by Hoshaw et al., [40] and was positively correlated to serotonin concentration in the hippocampus that demonstrated decrease in hippocampal serotonin level on administration of JB1, a selective pIGF-1R inhibitor to rat brain. Studies by Aguado et al. [41] showed presence of IGF-1 receptors on cytons of serotonergic neurons in the raphe that were co-localized in the hippocampus with projections from the raphe, thus possibly leading to the direct activation of serotonin release. Similar IGF-1 mediated mechanisms could have contributed to the increased serotonin concentration in the hippocampus on introduction of novel objects. Administration of PCPA to rats of SHNOPCPA group on the other hand, did not alter expression of pIGF-1R significantly indicating that serotonin had no role in IGF-1R phosphorylation in the hippocampus.

Since IGF-1 has been previously reported to mediate CREB phosphorylation which in turn leads to synaptic strengthening [42], we estimated CREB phosphorylation during the present study. Both SH and PH groups showed decrease in pCREB expression corresponding to decrease in the apical dendrite arborization. The preservation of dendritic architecture in the SHNO group on the other hand was concomitant to increase in pCREB expression.

## Conclusion

To summarise our findings, we demonstrate that monotony could induce anxiety and depression like traits in animal models independent of isolation. Interrupting monotony through introduction of novel objects during isolation to ameliorates anxiety and depression like traits and preserves neuronal architecture through an IGF-1-serotonin mediated mechanism. Novel object intervention not only increases IGF-1R phosphorylation but also results in increased phosphorylation of CREB and increase in serotonin concentration in the hippocampus that synergistically contribute towards the amelioration of anxiety and depression like traits during monotony stress. Further investigations on the mechanisms related to IGF-1R phosphorylation and neuronal circuits being activated on introduction of novel objects during monotony may provide valuable information for development of strategies for treatment of lifestyle and adjustment disorder related mood state alteration.

## Abbreviations

HPLC: High performance liquid chromatography
OFT: Open field test
EPM: Elevated plus maze test
FST: Force swim test
PCPA: Para-chloro phenyl alanine
i.p.: intraperitoneal
pIGF-1R: phospho-insulin like growth factor-1 receptor
pCREB: phospho cyclic AMP response-element binding protein
5-HT: 5-hydroxy tryptamine
5-HIAA: 5-hydroxy indole acetic acid
PHNO: Paired housed rats with novel objects intervention
PH: Paired housed rats
PHNOPCPA: Paired housed rats with novel objects intervention and PCPA administration
PHPCPA: Paired housed rats with PCPA administration
SH: Singly housed isolated rat
SHNO: Singly housed isolated rat with novel objects intervention
SHNOPCPA: Singly housed isolated rat with novel objects intervention and PCPA administration
